# Effects of forest wildfire on inner-Alpine bird community dynamics

**DOI:** 10.1371/journal.pone.0214644

**Published:** 2019-04-24

**Authors:** Livio Rey, Marc Kéry, Antoine Sierro, Bertrand Posse, Raphaël Arlettaz, Alain Jacot

**Affiliations:** 1 Division of Conservation Biology, Institute of Ecology and Evolution, University of Bern, Bern, Switzerland; 2 Swiss Ornithological Institute, Sempach, Switzerland; 3 Swiss Ornithological Institute, Valais Field Station, Sion, Switzerland; Ecole Pratique des Hautes Etudes, FRANCE

## Abstract

As major disturbance agents, natural catastrophes impact habitats, thereby maintaining the dynamics of ecological communities. Such discrete events are expected to positively affect biodiversity because they generate high habitat heterogeneity and thus numerous ecological niche opportunities. Species typical of open and semi-open habitats, which are often of high conservation concern in modern anthropized landscapes, may benefit most from recurrent natural catastrophes that regularly reset ecosystems. We investigated bird community changes and species-specific responses to wildfire at two recently burnt temperate, montane-subalpine forest stands in an inner-Alpine Swiss valley, with a special focus on red-listed and conservation priority species. We compared bird community changes in burnt forests (spanning 13 years) with bird assemblages occurring in adjacent non-burned forest stands that served as quasi-experimental controls. Strong species-specific responses to wildfire were evidenced, resulting in a dramatic post-fire decrease in overall bird abundance and species richness. Yet, red-listed bird species and conservation priority species in Switzerland were substantially more common in burnt than in control forest stands. Many red-listed species showed a bell-shaped numeric response to wildfire over time, suggesting low habitat suitability just after fire, high habitat suitability at pioneer and early stages of vegetation succession, followed by a long-term decrease in suitability while vegetation becomes denser, especially at ground level. As established for Mediterranean regions where wildfires are especially frequent, this study shows that forest fires can also boost the populations of red-listed and priority bird species typical of open and semi-open habitats in temperate biomes. Prescribed forest fire might represent a management option for preserving threatened elements of biodiversity despite the intense public debate it will trigger.

## Introduction

Most ecosystems are in essence dynamic natural systems in which biological community structure and population densities are modulated by ever changing environmental conditions that generate spatial heterogeneity [1], creating various ecological niche opportunities, notably via variegated successional vegetation stages [2]. Shifts in ecological communities can result either from environmental changes that progressively model habitat or from sudden catastrophes that disrupt the ecosystem functioning [3]. The latter are commonly defined as disturbances [3] and include, among others, fires, floods, landslides, avalanches, storms, droughts and tidal waves (e.g. [1]). However, generalisation about the magnitude of the impact of disturbances on communities is hampered by the huge variability observed in intensity, frequency and spatial extent of such events, which often define intricated environmental gradients whose components are difficult to disentangle [4].

Wildfires are an important agent of natural disturbance. Fires are discrete, unpredictable events that reduce living organisms, i.e. complex assemblages of molecules, into a few organic and mineral compounds [5–7]. Yet, at the same time, fire typically increases ammonium concentration in the soil, which boosts re-colonization by plants [5]. Moreover, tree carcasses persist in the form of trunks and limbs lying on the ground or snags ([8–11]; but see [12]), generating novel vertical and horizontal structural heterogeneity. The post-fire re-colonization by pioneer plant species causes a rapid rejuvenation of the ground and field layer, but leaves numerous patches of bare ground. Taken together, this creates a patchy mosaic of semi-open habitats [13]. Such a mosaic can offer optimal conditions for ground-feeding insectivorous birds, especially as bare ground enhances food availability, i.e., prey abundance modified by accessibility [14]. Studies describing the impact of wildfire on avifauna of grasslands and savannahs (e.g. [12, 15–17]), boreal and temperate forests (e.g. [11, 18–21]), tropical rainforests (e.g. [9, 22–23]) and Mediterranean ecosystems (e.g. [24–30]) mostly indicate shifts in communities towards open-land or non-forest bird species. This apparent deterministic change in the bird assemblage is likely to favour beta-diversity because burnt and unburnt patches often occur side by side, sometimes even in an intricate manner.

Forest wildfires are usually combatted all over the world not only to protect human settlements and timber resources, but also to avoid apparent harm for flora and fauna [31]. As a result, pioneer forest habitats have become rarer, and with them many of the threatened open-habitat species that they typically harbour, notably in the Mediterranean (e.g. [26–27, 29]) and in North America (e.g. [2, 15]). This has important implications for bird conservation. Besides the short-term changes towards open-land, non-forest species, long-term studies indicate that the species pool remains dissimilar in formerly burnt forest plots compared to unburnt plots for quite long periods [32], showing a species shift from very diverse communities of ground- and aerial-foraging birds towards more homogenous assemblages in old burnt forest stands [32]. Bird species composition is thus less stable in burnt areas than in areas that were not burnt recently, revealing a succession in species composition that resembles vegetation succession.

In this study, we investigated the community and species-specific responses of birds to wildfires occurring in continental, inner-Alpine woodland [33]. Wildfires occur recurrently in southern Switzerland (10% natural phenomenons, 90% human causes [34]) where they may play an important role in ecosystem dynamics. Their effects on ecological communities have been studied with strong focus on floral ([33, 35]) and arthropod communities ([36–38]). Here we focused on the effects of fire on birds with a specific emphasis on Swiss red-listed, including near-threatened, [39] and Swiss conservation priority bird species, hereafter priority species [40]. Our study design combines longitudinal data on the temporal avifauna trends in burnt montane and subalpine forests as well as cross-sectional data on birds of burnt *vs* adjacent intact control forest stands.

## Material and methods

### Study sites

We conducted this study in the burnt forests above Leuk (46°20’ N / 7°38’ E) and Visp (46°17’ N / 7°53’ E) and the adjacent unburnt control forests flanking them in the East and West. No permit was required to conduct the study. Both study areas are situated in SW Switzerland (Valais), the driest area of the Alps (ca. 550 mm rainfall pro annum in Sion). Valais is an East-West oriented inner-Alpine valley characterized by strong climate continentality [34]. In Leuk, the burnt forest extends from 850 to 2100 m a.s.l. on the south-facing slope above the Rhone River and covers ca 300 ha. The vegetation gradient changes with altitude from oak-pine formations (*Quercus pubescens*, *Pinus sylvestris*) at the bottom to spruce (*Picea abies)* and larch stands (*Larix decidua*) at the timberline [33]. The wildfire above Leuk took place on the 13^th^ of August 2003, in the middle of a heat wave with extremely dry conditions. It was the biggest forest fire recorded in Valais over a century, and the second largest ever in the inner-Alps [34]. Fire severity was high in large parts of the study site. Most plants were dead above ground, most of the organic layer of the soil was destroyed and trees were scorched to the top with no remaining foliage or fine twigs on standing trees [33, 35]. Apart from a roughly 1 ha large patch with intact trees, tree vegetation cover was only 1.5% one year after the fire and 3.6% 10 years after the fire [41]. For a detailed overview of the vegetation succession of the burnt forest in Leuk see [41]. The fire of Visp occurred on the 26^th^ of April 2011. The burnt area extends from 600 to 1200 m a.s.l. and covers ca 100 ha. It is situated on the north-facing slope above the Rhone River, ca 19 km East of Leuk. The vegetation gradient differs markedly from the site in Leuk. Both control forests are dominated by coniferous trees with a lack of deciduous trees. Again, fire severity was high (i.e. many standing dead trees and lying dead wood), while no detailed scientific information is available. In the past century around 60 wildfires affecting more than 5 ha each have been recorded in the canton of Valais, with a mean size of 26 ha, an average area that would correspond to small fires in the Mediterranean [34].

### Bird monitoring

All bird counts were performed along well defined transects following standard monitoring protocols of the Swiss Ornithological Institute [42], a so-called “simplified territory mapping”. With this method, every transect was surveyed three times between the 15^th^ of April and the end of June. Surveys were conducted between sunrise and 11 am at the latest and only on days without strong rain or wind. The transect starting point was always alternated between surveys of the same transect and sampling effort was kept constant irrespective of habitat and bird abundance.

The two burnt forests were surveyed along one transect each that crossed the entire burnt area. In Leuk, these surveys were conducted in 2006, 2007, 2008, 2010, 2012, 2014 and 2016, i.e. 3, 4, 5, 7, 9, 11 and 13 years after the fire. In Visp, they were conducted annually in 2012–2016, i.e. 1–5 years after the fire (see S1 Fig for a map with transects and S1 Table for monitoring dates). This dataset, which included only the surveys within the burnt zones, is hereafter called SOI (for Swiss Ornithological Institute) data.

To serve as a control for the effects of wildfire on the local bird communities, we additionally surveyed birds in adjacent unburnt forests (called control forests hereafter) in 2014 along systematically spaced line transects (see S2 Fig for a map with transects and S1 Table for monitoring dates). One transect always consisted of three subtransects, one in the burnt forest and one each in the control forest to the West and East of the burnt forest. Transects roughly followed altitudinal isolines and whenever possible used existing infrastructure such as roads and trails. We set up eight continuous transects in Leuk, where burnt and control forests were situated directly next to each other and four disjoint transects in Visp, where burnt and control forests were within 2 km of the burnt area.

### Delineation of bird territories

We assessed breeding bird territories in burnt and control forests by counting every observation of each species. The approximate location of every observation was noted on a satellite map of the respective area. We then transferred all observations into QGIS version 2.18.13 [43] and in these stacked surveys in a first step grouped clustered observations over the three surveys into territories for each species. In a second step, the remaining observations were as well allocated to territories. The final territory delineation was controlled by the person responsible for the monitorings of the burnt forests in previous years to ensure equal territory delineation over all years. Within QGIS, we then computed a 100 m buffer around each subtransect and for the analyses only used the territories with observations within the 100 m buffer. This resulted in one value (number of territories) per species and transect for the burnt forest and one value each for the control forests West and East of the burnt forest. We accounted for different lengths of subtransects by extrapolating the number of found territories per subtransect to10 ha. However, as we were interested in overall changes and not per transect, we summed up the found territories to get one value for the burnt forest and one value each for the control forests West and East of the burnt forest (e.g. for Eurasian Chaffinch in Leuk 2014: 7 territories burnt forest, 121 western control forest, 109 eastern control forest).

For the SOI data, we used a similar approach: as in all years except 2014 the data collection was conducted on one continuous transect criss-crossing the burnt areas, a direct comparison with the data collection of 2014 was not possible. We therefore laid the subtransect buffer from 2014 over the SOI data and selected only territories with observations within the buffer, i. e. the observations that would have been found walking on the transects walked in 2014. This resulted in territory counts for each species in the burnt forest for all monitored years and for the control forests in the year 2014. Corvid species and all birds of prey were excluded from the analysis because they use much larger areas, which means that their site use was affected by the burn/control distinction only incompletely ([29], see S2 Table).

In order to estimate breeding bird territories for the control forests from 2006–2016, we linked our 2014 data with the Swiss Bird Index (SBI). The SBI estimates the Swiss-wide species-specific yearly population size based on national survey data since 1990 [44]. Using 2014 as a baseline, we applied the correction factor based on this Swiss-wide monitoring scheme for our 2014 dataset and obtained territory estimates for each species and year, rounded to the nearest integer. However, we did not use the Swiss-wide index as a correction factor, but accounted only for survey data collected in the canton of Valais. This method assumes little or no changes in habitat structure within control forests but cannot account for year-dependent spillover effects of birds from the burnt forest into control forests.

We had originally intended to use distance sampling for the modeling and estimation of bird transect- and site-specific densities while accounting for imperfect detection [45–46]. However, we encountered very frequent problems with numerical convergence when trying to fit these models in the R software *Unmarked* [47]. Hence, we had to abandon that approach and instead used more traditional statistical analyses (see below) that ignore imperfect detection and thus only make inferences about the combination of abundance and detection. Our conclusions therefore are either for absolute numbers of territories or for "relative abundance", i.e. that detection probability does not vary systematically along our dimensions of comparison [46].

### Disturbance index calculation

To express the effects of wildfires on red-listed and priority species, we used a simple, standardized disturbance index that reflects the difference in the number of breeding bird territories between the burnt and the control forests for each year. Using this species-specific disturbance index we can additionally investigate the effects of wildfires on groups of species (e.g. Swiss red-listed or priority species). We expressed the index as follows:

$$\text{Disturbance}\;\text{Index}=\frac{n\_burnt-n\_control}{\sqrt{\frac{n\_burnt+n\_control}{2}}},$$

where n__burnt_ is the species-specific number of territories per 10 ha in the burnt forest and n__control_ is the species-specific number of territories in the control forest per 10 ha. This index has a positive value if a species has more territories in the burnt forest than in the control forest and a negative value if vice versa.

### Statistical analysis

Statistical analyses were conducted in R version 3.2.2 [48]. To analyse species-specific responses of the relative abundance to fire we used generalized linear mixed models (poisson distribution, package *lme4*, [49]) with species-specific territory counts as a dependent variable, forest state (burnt vs control forest) as fixed factor and years after fire as a covariate. Study site (Leuk vs Visp) entered the analysis as random factor. We always tested for quadratic effects (years after fire) and all 2-way interactions between forest state and years after fire. As model selection approach we used the same model structure for every species and identified the most parsimonious models according to lowest AICc with the dredge function (package MuMIn, [50]). For every species we retained all models within 2 AICc units of the best model as competing best models. When we found such tied models, we averaged over these models. Due to a very small sample size for some species, we evaluated competing best models for 37 species only (31 least concern, 5 near-threatened, 1 vulnerable species [39]; 5 Swiss priority species and 32 non-priority species [40] out of the total of 58 species.

Besides the species-specific analyses, we also analysed apparent species richness and overall relative abundance (with inferences again requiring the index assumptions, see above). As in the species-specific models we retained all competing models within 2 AICc units of the best model and, in case we found competing best models, model-averaged these best models. To compare species richness between the burnt forests and the control forests of each study site we also used generalized linear-mixed models with poisson distribution. Note that we always compared one burnt forest (Leuk or Visp) with two control forest stands (West and East of the burnt forest). We used the same approach to analyse bird abundance, but used linear mixed effect models. We estimated density by counting the number of territories per burnt or control forest within the buffer and extrapolated the result for 10 ha. When analysing the effect of fires on species richness and bird density we treated these values as the response, while forest state, years after fire (linear and squared) and all 2-way interactions between forest state and years after fire were our explanatory variables. Finally, study site was our random factor. We used the same model selection approach as for the species-specific approach to identify the most important factors affecting species richness and abundance.

To analyse the species responses to fire in relation to their Red list category (least concern, near-threatened and vulnerable) and Swiss conservation priority status we used two different statistical approaches. First, we used linear mixed effect models and used the overall species-specific disturbance index as a response variable and as explanatory variables: Red list category or Swiss priority status, years after fire (linear and squared) and all 2-way interactions between Red list category or Swiss priority status and years after fire. Finally, species and study site were our random factors. Second, we used generalized linear mixed models with poisson distribution with the actual species-specific number of territories as a response and forest state, Red list category or Swiss priority status and years after fire (linear and squared) as explanatory variables, along with the 2-way interactions between Red list category or Swiss priority status and forest state or years after fire and all 3-way interactions between Red list category or Swiss conservation priority status, forest state and years after fire. Species and study site were used as random factors. In contrast to the species-specific analyses, we included all species in these latter analyses.

## Results

In total we found 5 233 territories of 58 species (see S3 Table). 20 (34.5%) species were only found in Leuk, 1 (1.7%) only in Visp and the remaining 37 (63.8%) species were shared between the two study sites. 14 species (24.1%) were on the Swiss Red list (9 near-threatened, 5 vulnerable), and 11 species (19%) were considered Swiss priority species. In most species, our data suggest little spill-over between burnt and control forests, e.g. 334 territories of Eurasian Chaffinch in control forests but only 28 territories in burnt forests in 2014 or 11 vs. 56 territories in control vs. burnt forests of Common Redstart in 2014.

### Species-specific reactions to fire

In 33 species out of the 37 species for which best models could be fitted, the variable “forest state” with the two categories “burnt” and “control” appeared. (see Table 1 for detailed results of every species and S4 Table for an overview of competing best models).

**Table 1:** Time-dependent effects of forest fire on bird species.

Species	Model factors	Estimate	Std. error	z-value
**Black Grouse**	Intercept	-4.90	2.13	2.31
	Forest state	-0.39	0.50	0.77
**Common Cuckoo**	Intercept	-0.87	0.13	-6.48
	Forest state	-0.73	0.16	-4.46
**Eurasian Wryneck**	Intercept	-6.81	1.29	5.26
	Forest state	-2.61	1.15	2.27
	Years after fire	1.42	0.31	4.59
	Years after fire^2^	-0.07	0.02	4.2
	Forest state^1^ years after fire	-0.21	0.16	1.32
	Forest state^1^ years after fire^2^	-0.01	0.01	1
**Eurasian Green Woodpecker**	Intercept	-3.62	2.47	-1.47
	Forest state	-1.99	0.33	-6.07
**Black Woodpecker**	Intercept	-0.47	0.27	-1.78
	Forest state	-1.12	0.16	-7.53
**Great Spotted Woodpecker**	Intercept	-0.81	0.13	-6.25
	Forest state	-0.64	0.16	-4.08
**Tree Pipit**	Intercept	-0.85	0.77	1.10
	Forest state	-1.34	0.12	11.46
	Years after fire	0.14	0.12	1.15
	Years after fire^2^	-0.01	0.01	1.89
**Black Redstart**	Intercept	-0.01	0.35	0.16
	Forest state	-3.93	0.76	5.17
	Years after fire	-0.10	0.04	2.52
	Forest state^1^ years after fire	0.17	0.08	2.05
**Common Redstart**	Intercept	0.15	0.33	0.45
	Forest state	-3.07	0.23	13.17
	Years after fire	0.07	0.07	0.95
	Years after fire^2^	-0.01	0.01	1.13
	Forest state^1^ years after fire	0.03	0.04	0.82
**European Robin**	Intercept	0.06	0.06	0.06
	Forest state	3.64	3.64	3.64
	Years after fire	-2.68	-2.68	-2.68
	Forest state ^1^ Years after fire	2.96	2.96	2.96
**Blackcap**	Intercept	-1.69	0.59	-2.85
	Forest state	1.6	0.44	3.62
	Years after fire	0.79	0.14	5.52
	Years after fire^2^	-0.06	0.01	-5.61
	Forest state * Years after fire	-0.67	0.15	-4.49
	Forest state * Years after fire^2^	0.05	0.01	4.95
**Western Bonelli's Warbler**	Intercept	-0.25	0.7	0.36
	Forest state	0.33	0.69	0.48
	Years after fire	-0.11	0.25	0.44
	Years after fire^2^	0.03	0.01	1.82
	Forest state^1^ years after fire	0.26	0.28	0.93
	Forest state^1^ years after fire^2^	-0.03	0.02	1.47
**Common Chiffchaff**	Intercept	-1.42	0.47	3.02
	Years after fire	0.13	0.12	1.08
	Years after fire^2^	-0.01	0.01	1.63
**Eurasian Blackbird**	Intercept	-0.02	0.14	0.15
	Forest state	-0.65	0.11	6.12
	Years after fire	0.03	0.02	1.6
**Ring Ouzel**	Intercept	-3.49	1.45	2.40
	Forest state	0.39	0.26	1.49
	Years after fire	0.03	0.03	1.22
**Song Thrush**	Intercept	-1.82	0.38	4.84
	Forest state	1.15	0.39	2.99
	Years after fire	0.03	0.05	0.69
	Forest state^1^ years after fire	0.07	0.06	1.2
**Mistle Thrush**	Intercept	-0.5	0.20	2.46
	Forest state	0.05	0.05	1.07
	Years after fire	-0.12	0.17	0.69
	Years after fire^2^	-0.01	0.004	1.08
	Forest state^1^ years after fire	-0.03	0.03	1.09
**Great Tit**	Intercept	-1.56	0.26	-6.06
	Forest state	0.03	0.31	0.09
	Years after fire	0.18	0.03	6.63
	Forest state ^1^ Years after fire	-0.16	0.04	-4.59
**Eurasian Blue Tit**	Intercept	-4.94	1.26	3.91
	Forest state	-0.50	1.58	0.32
	Years after fire	0.25	0.12	2.12
	Forest state^1^ years after fire	-0.23	0.16	1.43
**Willow Tit**	Intercept	-0.22	0.1	-2.25
	Forest state	-0.34	0.11	-3.06
**Coal Tit**	Intercept	0.34	0.24	1.42
	Forest state	0.90	0.23	3.96
	Years after fire	-0.07	0.03	-2.05
	Forest state ^1^ Years after fire	0.1	0.03	2.96
**Crested Tit**	Intercept	-1.6	0.22	7.23
	Forest state	1.9	0.21	8.98
	Years after fire	-0.02	0.01	1.68
**Long-tailed Tit**	Intercept	-3.03	0.54	5.6
	Years after fire	0.15	0.25	0.6
	Years after fire^2^	-0.03	0.02	1.49
**Winter Wren**	Intercept	-0.56	0.52	1.08
	Forest state	-1.28	0.43	2.99
	Years after fire	0.26	0.16	1.66
	Years after fire^2^	-0.03	0.01	2.29
	Forest state^1^ years after fire	0.01	0.01	2.85
	Forest state^1^ years after fire^2^	0.18	0.07	2.58
**Dunnock**	Intercept	-4.46	5.12	0.87
	Forest state	-0.66	0.39	1.68
	Years after fire	-0.12	0.1	1.25
	Years after fire^2^	-0.01	0.01	1.29
	Forest state^1^ years after fire	0.17	0.05	3.16
	Forest state^1^ years after fire^2^	0.01	0.004	3.13
**Wood Nuthatch**	Intercept	-1.27	0.78	1.64
	Forest state	-0.6	0.64	0.93
	Years after fire	-0.14	0.22	0.63
	Years after fire^2^	-0.02	0.02	1.29
	Forest state^1^ years after fire	0.31	0.12	2.64
	Forest state^1^ years after fire^2^	0.03	0.01	2.34
**Eurasian Treecreeper**	Intercept	-1.76	1.03	1.71
	Forest state	0.24	1.02	0.24
	Years after fire	0.39	0.35	1.12
	Years after fire^2^	-0.05	0.03	1.7
	Forest state^1^ years after fire	0.04	0.03	1.29
	Forest state^1^ years after fire^2^	-0.53	0.36	1.46
**Eurasian Jay**	Intercept	-1.70	0.33	-5.23
	Forest state	0.25	0.37	0.69
	Years after fire	0.11	0.04	3.05
	Forest state ^1^ Years after fire	-0.1	0.04	-2.29
**Spotted Nutcracker**	Intercept	-2.95	0.38	-7.79
	Forest state	0.8	0.4	2.01
**Eurasian Chaffinch**	Intercept	0.77	0.17	4.46
	Forest state	0.46	0.16	2.86
	Years after fire	-0.06	0.04	1.74
	Years after fire^2^	0.09	0.02	3.87
	Forest state^1^ years after fire	-0.003	0.002	1.63
**European Goldfinch**	Intercept	-1.27	0.83	1.54
	Forest state	-1.35	0.76	1.78
	Years after fire	-0.03	0.16	0.19
	Years after fire^2^	-0.01	0.01	1.05
	Forest state^1^ years after fire	0.17	0.08	2.11
**Alpine Citril Finch**	Intercept	-2.97	4.90	0.61
	Forest state	-1.36	0.34	3.96
	Years after fire	-0.50	0.16	3.23
	Years after fire^2^	0.02	0.01	2.29
	Forest state^1^ years after fire	0.08	0.05	1.58
	Forest state^1^ years after fire^2^	0.01	0.003	1.56
**Eurasian Linnet**	Intercept	-4.1	3.17	1.29
	Forest state	-1.13	0.26	4.32
	Years after fire	-0.04	0.04	1.04
**European Serin**	Intercept	0.2	0.46	0.43
	Forest state	-4.69	0.87	5.38
	Years after fire	0.05	0.18	0.28
	Years after fire^2^	-0.03	0.02	1.97
	Forest state^1^ years after fire	0.53	0.11	4.8
	Forest state^1^ years after fire^2^	0.04	0.01	4.28
**Eurasian Bullfinch**	Intercept	-0.78	0.2	3.9
	Forest state	-0.49	0.16	3.15
	Years after fire	-0.02	0.02	0.95
**Red Crossbill**	Intercept	-5.25	1.65	3.18
	Forest state	2.51	0.54	4.63
	Years after fire	0.67	0.23	2.31
	Years after fire^2^	-0.04	0.02	2.42
**Rock Bunting**	Intercept	0.58	0.63	0.92
	Forest state	-3.03	0.20	15
	Years after fire	0.09	0.16	0.56
	Years after fire^2^	-0.02	0.01	1.73

For species with competing best models in terms of AIC, models have been averaged. For a model overview of species with competing best models, see S4 Table.

^1^ For the factor “forest state”, a negative coefficient indicates a higher abundance in burnt forests compared to control forests.

^2^ For the factor “years after fire”, a negative coefficient indicates a decrease in abundance with years after fire.

**Fig 1:**
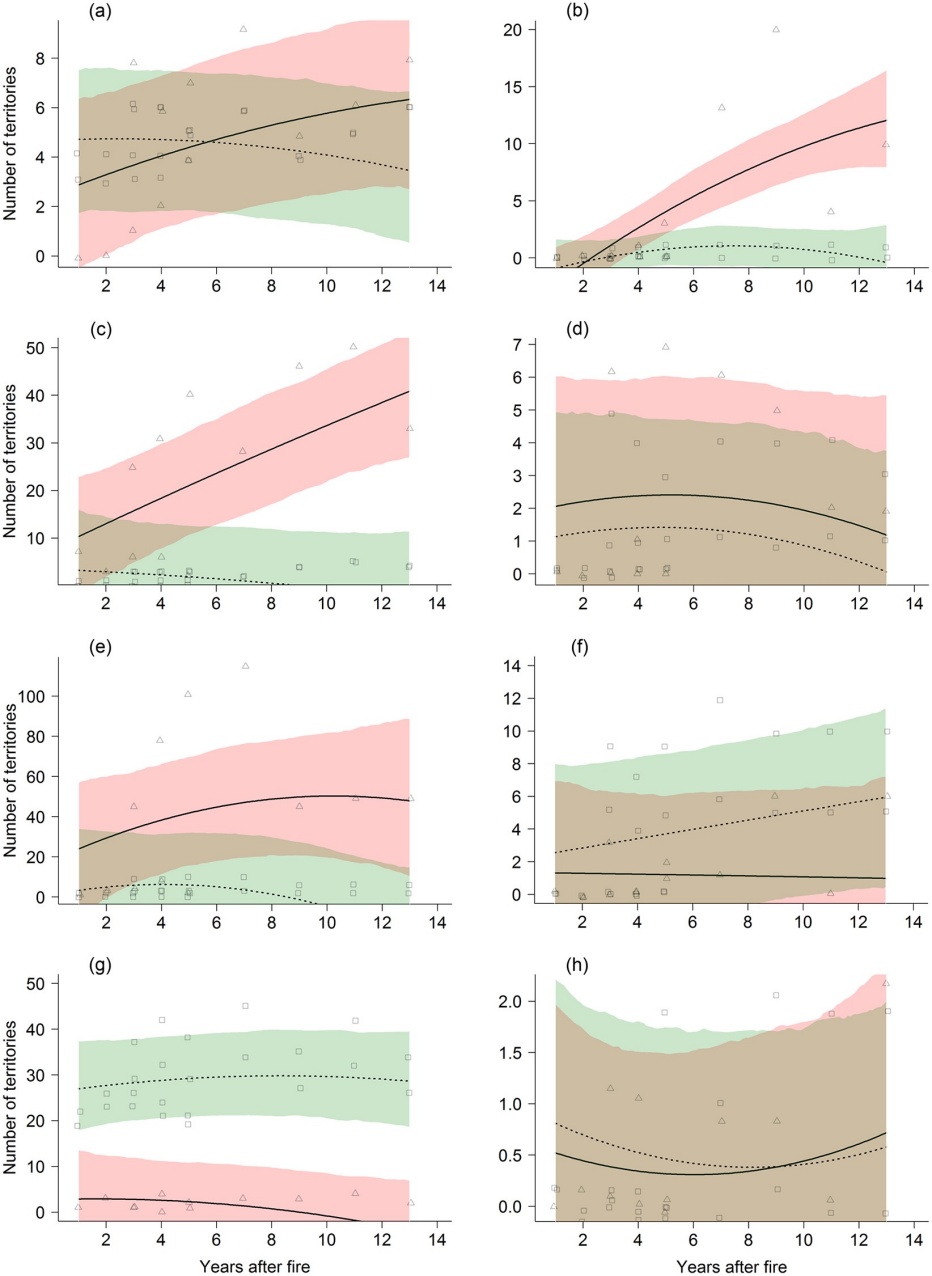
Number of territories in relation to years after fire. Shown are regression lines and 95% confidence intervals for the burnt forest (solid line, red) and control forest (dashed line, green) for Common Cuckoo (a), Eurasian Wryneck (b), Common Redstart (c), Eurasian Linnet (d), Rock Bunting (e), Ring Ouzel (f), Crested Tit (g), and Black Grouse (h).

Fourteen species (Common Cuckoo (Fig 1a), Eurasian Wryneck (Fig 1b), Eurasian Green Woodpecker, Black Woodpecker, Great Spotted Woodpecker, Tree Pipit, Common Redstart (Fig 1c), Eurasian Blackbird, Willow Tit, Eurasian Blue Tit, Eurasian Linnet (Fig 1d), Eurasian Bullfinch, Alpine Citril Finch and Rock Bunting (Fig 1e)) were significantly more abundant in burnt forests than in the adjacent control forests. The Rock Bunting for example is a species that greatly benefitted from wildfires (forest state: z = 14.99, df = 4, p < 0.001, see Fig 1e) irrespective of the time after fire. Three of these species increased significantly over time in the burnt forest (linear: Common Redstart, Eurasian Blue Tit, squared: Eurasian Wryneck), while a fourth species (Alpine Citril Finch) decreased significantly after wildfire.

Five species (Ring Ouzel (Fig 1f), Song Thrush, Crested Tit (Fig 1g), Spotted Nutcracker and Red Crossbill) were significantly more abundant in control forests than burnt forests. For example the Crested Tit declined in the burnt forests after fire (forest state: z = 9.31, df = 3, p < 0.001, see Fig 1g). Of these species, only the Song Thrush increased in numbers over the years.

In fourteen species, the effect of the wildfire was more complex and the number of territories also depended on years after the disturbance (linear: Black Redstart, European Robin, Great Tit, Coal Tit, Dunnock, Wood Nuthatch, Eurasian Jay, Eurasian Chaffinch, European Goldfinch and European Serin, squared: Blackcap, Western Bonelli’s Warbler, Winter Wren and Eurasian Treecreeper). Nine of these species decreased significantly over time in burnt forests while no such trend was observed in control forests (linear: European Robin, Coal Tit, Dunnock, Wood Nuthatch, Eurasian Chaffinch, European Goldfinch and European Serin, square: Winter Wren and Eurasian Treecreeper). Two species (linear: Great Tit and Eurasian Jay) showed the opposite trend, they increased in burnt forests while no such trend was observed in control forests. The Blackcap and the Western Bonelli’s Warbler were more abundant in control forests but showed different trends: whereas Blackcap showed a bell-shaped relationship with maximum number of territories in both burnt and control forests 8–10 years after fire, Western Bonelli’s Warbler showed a quadratic increase both in burnt and control forests.

In four species (Black Grouse (Fig 1h), Mistle Thrush, Common Chiffchaff and Long-tailed Tit) the covariate forest state did not feature in the best model. The Mistle Thrush increased over time irrespective of forest state, the best model for Black Grouse, Common Chiffchaff and Long-tailed Tit was the null model with a constant only.

### Effects of fires on observed bird species richness and relative abundance

The average number of species in burnt and control forests depended on forest state irrespective of any other variable. Observed species richness did not differ significantly between burnt and control forests, z = 0.24, df = 3, see S5 Table).

**Fig 2:**
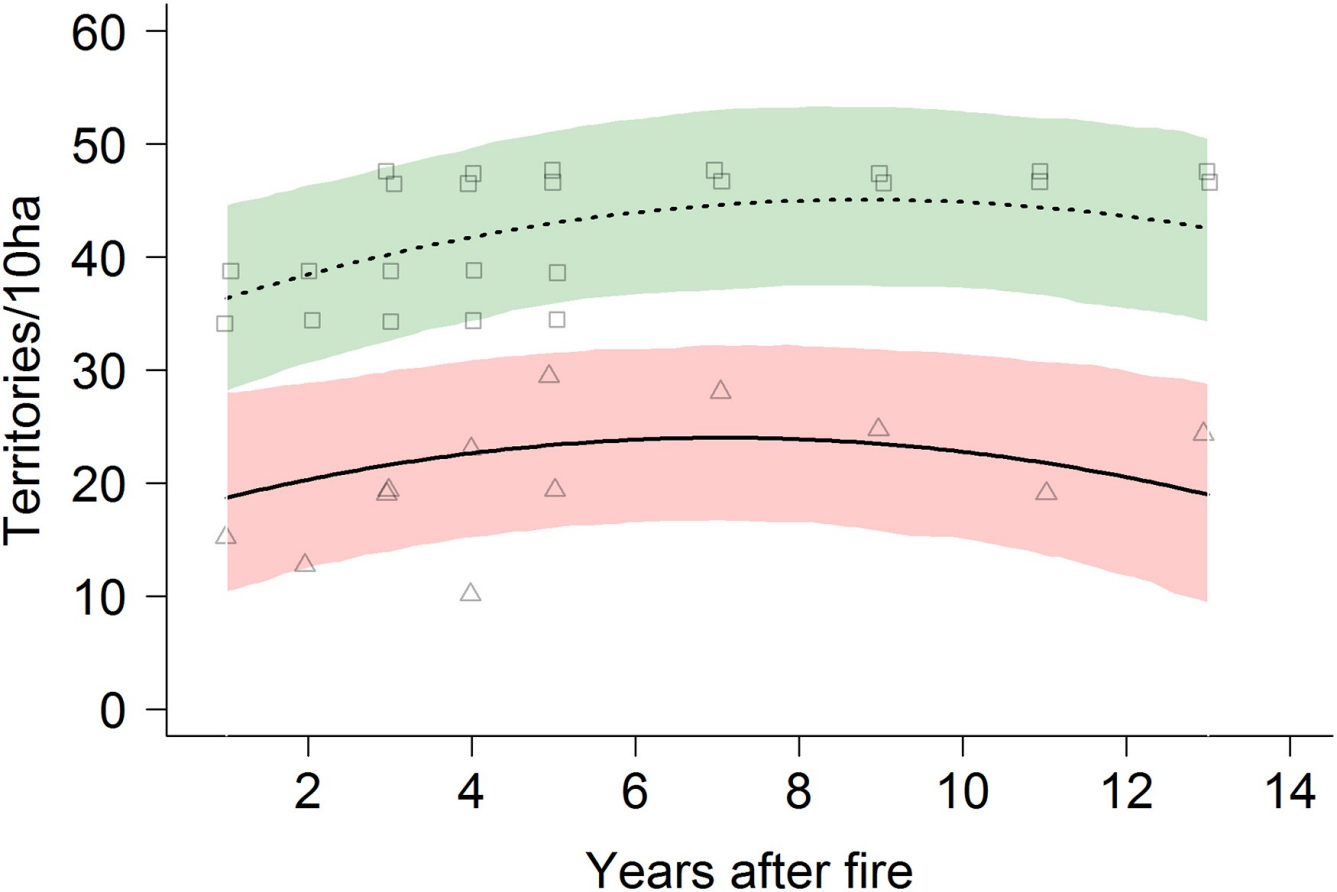
Average number of territories per 10 ha over all species in relation to years after fire. Shown are regression lines and 95% confidence intervals for the burnt forest (solid line, red) and control forest (dashed line, green).

In contrast to species richness, the number of territories per 10 ha, was significantly higher in control forests (41.59 ± 1.4) than in burnt forests (21.68 ± 5.0, t = 14.04, df = 4, p < 0.001, see Fig 2 and S6 Table).

### Effects of fires on red-listed and priority bird species

**Fig 3:**
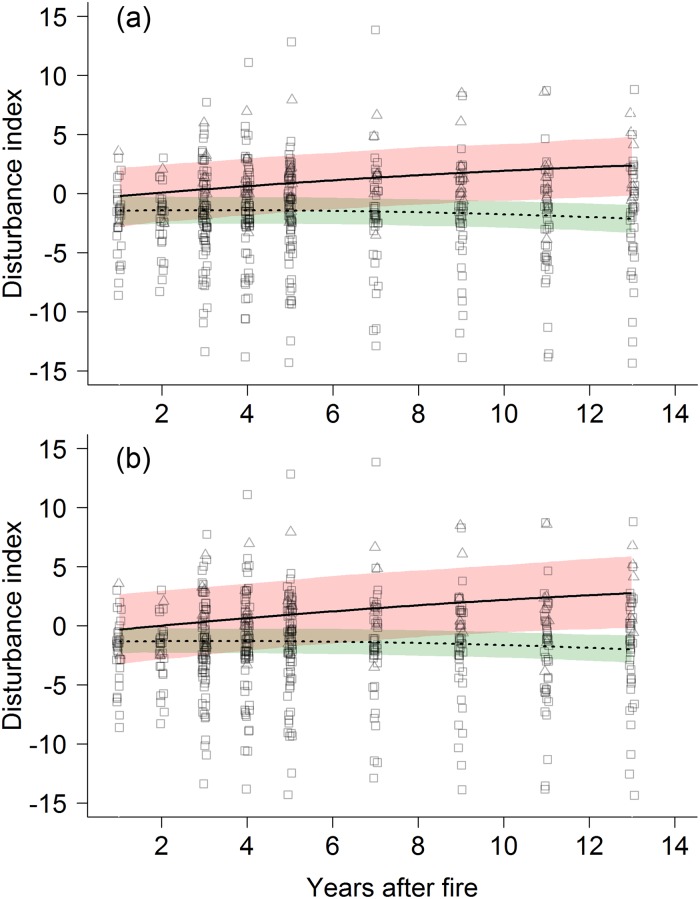
Disturbance index values in relation to years after fire. Shown are regression lines and 95% confidence intervals, for (a) Swiss red-listed bird species, where red = higher red-listed species, green = least concern species, and (b) Swiss priority bird species, where red = Swiss priority species, green = non-priority species.

Overall, red-listed species showed positive disturbance indices indicating that they were more common in burnt than in control forests. This contrasts with non-red listed species that suffered from a wildfire, highlighted by the negative disturbance indices. The effect of a wildfire on red-listed species was overall positive and became even stronger with years after fire (interaction Red list category * years after fire: z = 3.67, df = 7, p < 0.001, see Fig 3a and Table 2). A similar effect was found for Swiss priority bird species—they benefitted from a fire and the effect became stronger with years after fire (interaction Swiss priority status * years after fire: t = 4.02, df = 7, p = 0.01, see Fig 3b and S6 Table).

**Table 2:** Model selection table for Red list analysis with index.

Intercept	Red list category	years after fire	Red list category* years after fire	df	logLik	AICc	delta
-1.18	+	-0.07	+	7	-1076.48	2167.2	0
-1.53	+			5	-1079.06	2168.2	1.04

* For an overview of the best model for Red list analysis with index after model averaging, see S7 Table.

When analysing the actual number of territories between red-listed and non red-listed species we detected a similar pattern. Among the 5 competing best models, we always detected the three-way interaction between Red list category, forest state and years after fire, either as a quadratic (Interaction Red list category* forest state * years after fire^2^: z = 2.35, df = 13, p = 0.02, see Table 3) or a linear relationship (interaction Red list category* forest state* years after fire: z = 2.38, df = 13, p = 0.02). These 3-way interactions highlight that red-listed species benefitted from wildfires, where the magnitude of positive effects depended on the years after fire. However, the quadratic relationship shows that this effect was only temporary. These results were confirmed by the analysis focusing on Swiss priority species where four competing best models remained. Both the quadratic (interaction Swiss priority status* forest state* years after fire^2^: z = 2.52, df = 12, p = 0.01, see Table 4) and the linear relationships of the 3-way interactions were significant and retained as competing best models in the model selection approach (interaction Swiss priority status* forest state* years after fire: z = 2.5, df = 12, p = 0.01). As well as for red-listed species, this result shows that priority species reacted positively to wildfires, in contrast to non-priority species. Again, the quadratic effect shows that this effect was only temporary.

**Table 3:** Model selection table for Red list analysis with territories.

Intercept	Forest-state	Red list category	years after fire	years after fire^2^	Rl*y	Rl*y	Rl* Fs	Fs y	Fs y	Rl* Fs y	Rl* Fs y	df	logLik	AICc	delta	weight
-2.6	+	+	0.04	-0.01	+		+	+		+		12	-4433.14	8890.40	0.00	0.32
-2.67	+	+	0.07	-0.01	+	+	+		+		+	13	-4432.84	8891.90	1.43	0.16
-2.72	+	+	0.08	-0.01		+	+		+		+	12	-4433.88	8891.90	1.48	0.15
-2.58	+	+	0.03	-0.01	+	+	+	+		+		13	-4432.90	8892.00	1.55	0.15
-2.64	+	+	0.05	-0.01	+		+	+	+	+		13	-4433.02	8892.20	1.80	0.13

* Rl = Red list category,

y = years after fire,

Fs = forest state. For an overview of the best model for Red list analysis with territories after model averaging, see S7 Table.

**Table 4:** Model selection table for priority analysis with territories.

Intercept	Forest-state	Priority status	years after fire	years after fire^2^	Pr*y	Pr*y	Pr*Fs	Fs y	Fs y	Pr*Fs* y	Pr*Fs* y	df	logLik	AICc	delta	weight
-2.8	+	+	0.04	-0.01	+		+	+		+		12	-4438.60	8901.40	0.00	0.29
-2.9	+	+	0.08	-0.01		+	+		+		+	12	-4438.70	8901.60	0.20	0.26
-2.88	+	+	0.07	-0.01	+	+	+		+		+	13	-4438.37	8902.90	1.56	0.13
-2.87	+	+	0.07	-0.01	+		+	+	+	+		13	-4438.45	8903.10	1.72	0.12

* Pr = Priority status,

y = years after fire, Fs = forest state. For an overview of the best model for priority analysis with territories after model averaging, see S7 Table.

## Discussion

This study shows that wildfires dramatically impact forest avifauna and provide niche opportunities for a wide palette of bird species that are red-listed and/or bear highest conservation priority in Switzerland. This is established for the first time for a Central European, temperate inner-Alpine valley, adding to a growing body of evidence stemming from other biomes such as the Mediterranean (e.g. [27, 29–30, 51–52]), boreal and temperate forests (e.g. [11, 20]) and grasslands (e.g. [2, 15]).

Overall, we found more territories in intact control forests than in burnt forests, but species richness did not differ significantly between burnt and control forests (e.g. [20, 51]). Birds encountered in the intact control forests were in majority non-priority species of least conservation concern. This is because montane and subalpine forests have vast extensions throughout Switzerland, i.e. large populations of typical forest birds. We only truly sampled bird species in control forests in 2014, which was then extrapolated to the other years with a correction factor of the highly standardized swiss-wide bird monitoring scheme. Even though our results therefore need to be interpreted with caution, the general pattern was striking: near-threatened, threatened and priority species were more frequently encountered in burnt forests than in control forests in 2014, suggesting that the ecological differences between burnt and control forests was fairly large. Moreover, many typical forest species were encountered only in very low numbers in burnt forests. Even 11 years after the wildfire of Leuk, when burnt and control forests started to be more similar than in the beginning, this difference was still well visible. The missing spill-over between forest types suggests that bird communities within control forests have not changed as much as between forest types. Additionally, Moretti et al. [53] found no change in arthropod species richness in unburnt forests next to the burnt forest of Leuk, suggesting that no major changes occurred in unburnt control forests. We therefore argue that despite all caution needed to interpret the results, our approach with a regional correction factor from monitoring data allows conclusions about general changes in the avifauna of control forests. Our findings correspond to many studies from the Mediterranean, stating that open-habitat and threatened bird species benefit from wildfires [27, 29–30, 51]. In contrast to these study sites, however, wildfires in Switzerland are much rarer and less extended than in the Mediterranean. Nevertheless, we found similar effects of fire on birds, suggesting that many species are not specialized in inhabiting burnt areas, while a few open-habitat species and ground-foraging species can definitely benefit from wildfires and colonise burnt areas.

Red-listed and priority bird species, which in Switzerland belong mostly to species of open and semi-open habitats, occurred in relatively hight numbers in the burnt areas but only in low numbers, if even present, in the adjacent unburnt forest stands. As major environmental disruptors, forest wildfires have thus positive effects on threatened bird species. This is the case at least during the first stages of vegetation succession, notably because the habitat mosaic generated enhances beta-biodiversity, essentially through the local admixture of species relying on contrasted habitat configurations, notably densely vegetated vs open habitat. Yet, the favourable time window of habitat suitability is species-specific and can be quite short, as demonstrated by several species that already appeared to have faced declines during the course of our study. Several bird species (Eurasian Wryneck, Tree Pipit and Rock Bunting) showed signs of levelling off around 12 years after fires, roughly matching findings from Spain (10–19 years; [54]). The bird species that typically thrive in the early stages of the succession were mainly insectivorous ground-foraging species that rely on an appropriate ground-vegetation layer to access their food source, as demonstrated for several farmland birds [55–56]. Species such as Eurasian Wryneck and Common Redstart, present in our burnt forests, need patches of bare-ground to chase their arthropod prey. Bare ground is likely to increase prey availability, which means that prey is not only abundant but also accessible for birds. Interestingly, in contrast to Eurasian Wryneck, Tree Pipit and Rock Bunting, the number of territories for Common Redstart have not yet started to decrease, suggesting that other, unsurveyed factors may also play a role, like the availability of nesting sites.

Another important habitat component of the burnt forest is the presence of snags, which offer *sine qua non* breeding opportunities, in particular for obligatory cavity-nesting bird species, to the point that cavity availability dictates both species richness and density [10]. All regularly encountered woodpecker species were significantly more numerous in burnt forests than control forests, which is in line with many other studies (e.g. [14, 57]). Interestingly, this was not found for other species with similar ecology, like Eurasian Treecreeper and Wood Nuthatch. The reason for this is unclear, but might be due to the absence of bark, where both species like to forage, or the absence of a forest canopy cover. Different effects of fire were also found on various Tit species: Willow Tit and Eurasian Blue Tit were more numerous in burnt forests than in control forests and Great Tit increased significantly in burnt forests, whereas Coal Tit decreased significantly in burnt forests and Crested Tit was more numerous in control forests. Coal Tit and Crested Tit very much depend on coniferous trees, which were nearly absent in burnt forests. However, Zozaya et al. [58] found an increase of Crested Tit after wildfires when trunks were available. Contrastingly, the other three Tits prefer a higher amount of deciduous vegetation and possibly benefitted from a high amount of pioneering deciduous trees (Silver birch *Betula pendula*, Common aspen *Populus tremula* and *Salix appendiculata/caprea*), that were the dominant tree species after the fire in Leuk [33, 41]. This might also be the explanation for different reactions of Thrushes to the fires. The Eurasian Blackbird was more numerous in burnt forests compared to control forests. More so than the Eurasian Blackbird, the Song Thrush is a forest-dweller and was therefore encountered in larger numbers in control forests than in burnt forests. The Mistle Thrush in contrast inhabits a wide variety of habitats from closed forests to open habitats, possibly for which reason the factor “Forest state” did not appear in the best model for Mistle Thrush.

The positive effects of wildfires on threatened bird species so far observed in Mediterranean, temperate and boreal biomes may not be generalised to all types of woodland. In tropical rainforests, for instance, fires fragment the habitat, ususally promoting generalist bird species of little conservation value [9, 22]. Moreover, even within naturally fire-prone ecosystems, the effects of fires on particular threatened open-land species might be negative, notably where fire recurrence frequency is augmented due to anthropogenic factors [59]. We can therefore not generalise about a positive effect of forest wildfire on biodiversity, its impact depending on environmental context. Already in this study, we found marked differences in bird species richness between the two burnt forests: More than one third of all bird species found occurred at Leuk only, but not in Visp. This result is likely due to the south exposure of Leuk and the greater extent (ca threefold) of the burnt area. Additionally, there was a much larger altitudinal gradient in Leuk than in Visp, leading to a wider palette of habitats.

Although restricted to two large fires only [60], this study is one of the first contributions to understanding bird community responses to forest fire in central Europe. From our findings, we suggest that prescribed fire might be an appropriate management option to promote red-listed and priority bird species in Switzerland. This has also been proposed (e.g. [61]), discussed (e.g. [62]) and experimentally tested (e.g. [63–64]) in biodiversity management in Scandinavia and in the Mediterranean [52]. However, this would certainly request a major paradigm shift in the way we conceive forest protection, which calls for massive information campaigns to educate the public about the importance of wildfire to maintain forest dynamics, i.e. a broad palette of ecological niche opportunities among woodland [2]. Initiatives and infrastructure that aim at systematically combatting forest fires should be publicly debated and the appropriateness of related investments democratically questioned. More generally, the public has to understand that environmental disturbances are an intrinsic property of all ecosystems and that they have shaped the co-evolutionary processes beyond ecological community structuring. Such disturbances have unfortunately become rare in our human-dominated landscapes [31], not only because of systematic fire suppression [65] and mitigation measures against wind throws in forest [66], but for instance also because of river regulation against flooding [2, 67].

## Supporting information

S1 Fig Map of Leuk (a) and Visp (b) with transects walked in all years for SOI data collection. One square is 1 km^2^. Reprinted from map.geo.admin.ch under a CC BY license, with permission from swisstopo (BA18049), original copyright 2018.
(TIF)

S2 Fig Map with predefined transect lines of Leuk (a) and Visp (b) walked by the first author in 2014. One square is 1 km^2^. Red = subtransect within the burnt forest, blue = subtransects within control forests. Note that transects are surrounded by the 100 m buffer. Reprinted from map.geo.admin.ch under a CC BY license, with permission from swisstopo (BA18049), original copyright 2018.
(TIF)

S1 Table Monitoring dates since monitoring is conducted, with transect details. (DOCX)

S2 Table List of excluded species in all years with Swiss Red list category and Swiss priority status. Several corvid species and all birds of prey were excluded from the analysis. LC = least concern, NT = near-threatened, VU = vulnerable, EN = endangered.
(DOCX)

S3 Table List of all species found in our study sites in all years with Swiss Red list category and Swiss priority status. Species highlighted in bold were used for species-specific analyses, while all species were used for community analyses (LC = least concern, NT = near-threatened, VU = vulnerable).
(DOCX)

S4 Table Species-specific best models calculated with the dredge function, for species with competing best models in terms of AIC. Numbers following a species name distinguish different but equivalent models in terms of AIC.
(DOCX)

S5 Table Model selection tables for analyses, where competing best models were found. Model selection table for species richness.
(DOCX)

S6 Table Model selection tables for analyses, where no competing best models were found. a Model selection table for species abundance. b Model selection table for priority analysis with index.
(DOCX)

S7 Table Averaged best models for Red list and priority analyses, calculated only for analyses with competing best models from Tables 2–4. a Best model for Red list analysis with index after model averaging. b Best model for Red list analysis with territories after model averaging. c Best model for priority analysis with territories after model averaging.
(DOCX)
