# Supplementary figures and images for: Effects of forest wildfire on inner-Alpine bird community dynamics
Source: PLoS One. 2019 Apr 24;14(4):e0214644. doi: 10.1371/journal.pone.0214644 (PMC6481801; doi:10.1371/journal.pone.0214644)

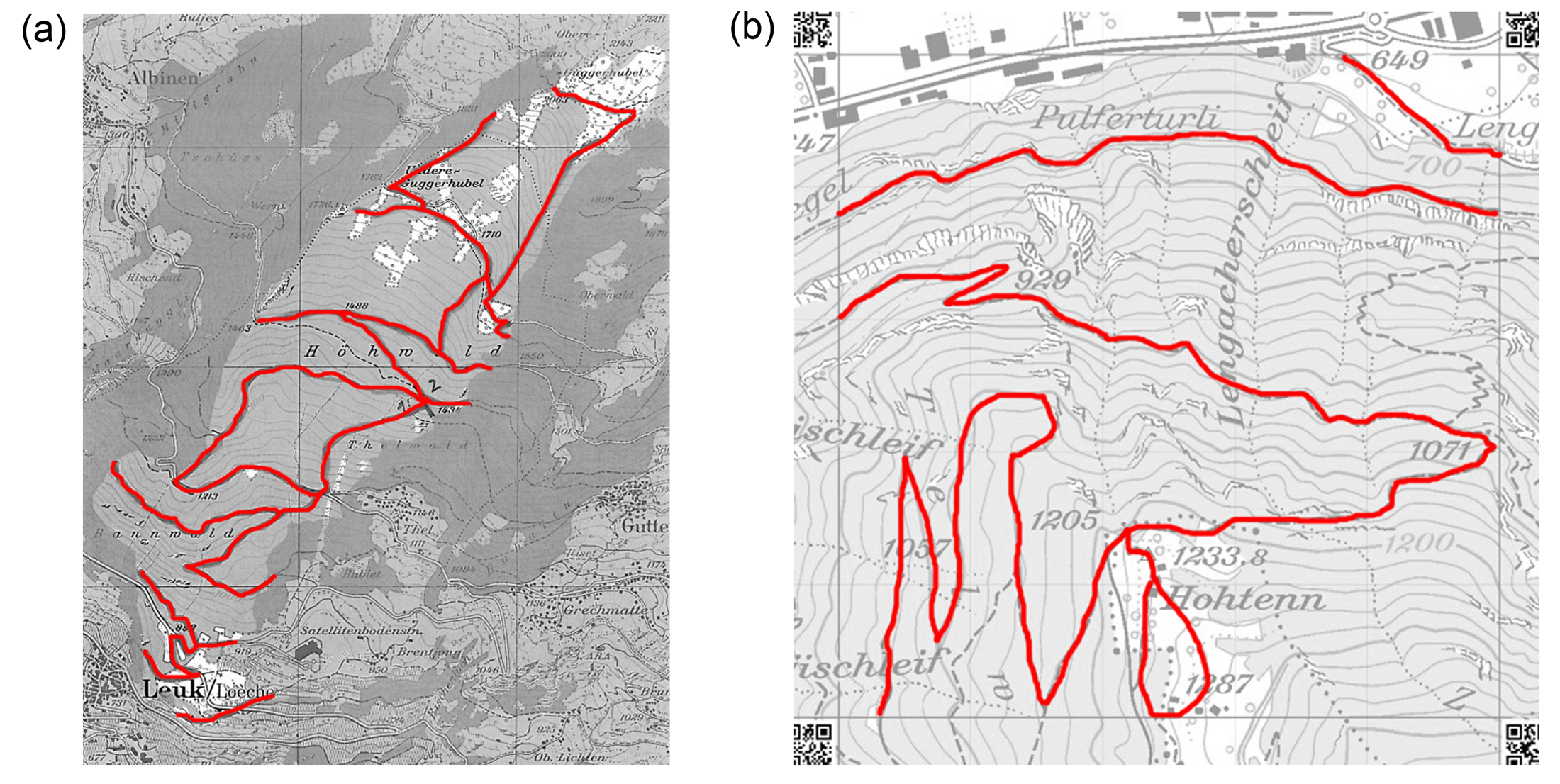

Supplement: S1 Fig — One square is 1 km2. Reprinted from map.geo.admin.ch under a CC BY license, with permission from swisstopo (BA18049), original copyright 2018. (TIF) [file pone.0214644.s001.tif]

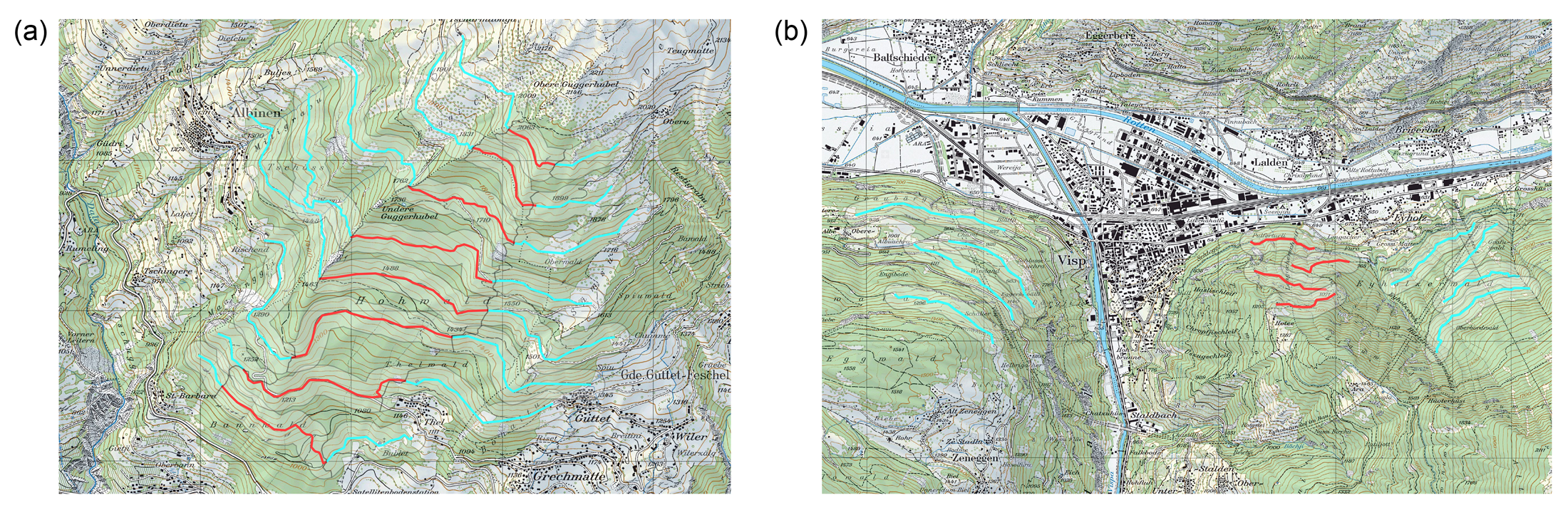

Supplement: S2 Fig — One square is 1 km2. Red = subtransect within the burnt forest, blue = subtransects within control forests. Note that transects are surrounded by the 100 m buffer. Reprinted from map.geo.admin.ch under a CC BY license, with permission from swisstopo (BA18049), original copyright 2018. (TIF) [file pone.0214644.s002.tif]
