# Supplementary material for: Effects of forest wildfire on inner-Alpine bird community dynamics
Source: PLoS One. 2019 Apr 24;14(4):e0214644. doi: 10.1371/journal.pone.0214644 (PMC6481801; doi:10.1371/journal.pone.0214644)
Supplement: S1 Table — (DOCX) [file pone.0214644.s003.docx]

**S1 Table. Monitoring dates since monitoring is conducted, with transect details.**

| **transect** | **site** | **year** | **monitoring date 1** | **monitoring date 2** | **monitoring date 3** | **length [m]** | **area [ha]** | **elevation [m]** |
| --- | --- | --- | --- | --- | --- | --- | --- | --- |
| - | Leuk | 2006 | 20.04. | 15.05. | 07.06. | - | - | - |
| - | Leuk | 2007 | 23.04. | 16.05. | 12.06. | - | - | - |
| - | Leuk | 2008 | 25.04. | 09.06. | 24.06. | - | - | - |
| - | Leuk | 2010 | 26.04. | 19.05. | 07.06. | - | - | - |
| - | Leuk | 2012 | 23.04. | 17.05. | 14.06. | - | - | - |
| - | Leuk | 2014 | 17.04 | 12.05. | 03.06. | - | - | - |
| - | Leuk | 2016 | 02.05. | 18.05. | 09.06. | - | - | - |
| - | Visp | 2012 | 28.04. | 24.05. | 07.06. | - | - | - |
| - | Visp | 2013 | 25.04. | 11.05. | 06.06. | - | - | - |
| - | Visp | 2014 | 26.04. | 15.05. | 01.06. | - | - | - |
| - | Visp | 2015 | 22.04. | 08.05. | 28.05. | - | - | - |
| - | Visp | 2016 | 20.04. | 16.05. | 27.05. | - | - | - |
| 1 | Leuk | 2014 | 26.04. | 16.05. | 07.06. | 1980 | 40.556 | 1060 |
| 2 | Leuk | 2014 | 26.04. | 16.05. | 07.06. | 2745 | 57.759 | 1230 |
| 3 | Leuk | 2014 | 25.04. | 17.05. | 05.06. | 3610 | 73.804 | 1360 |
| 4 | Leuk | 2014 | 03.05. | 22.05. | 09.06. | 3660 | 75.446 | 1450 |
| 5 | Leuk | 2014 | 23.04. | 20.05. | 10.06. | 3160 | 64.243 | 1530 |
| 6 | Leuk | 2014 | 24.04. | 21.05. | 11.06. | 3100 | 63.410 | 1750 |
| 7 | Leuk | 2014 | 04.05. | 24.05. | 12.06. | 2160 | 45.338 | 1870 |
| 8 | Leuk | 2014 | 04.05. | 24.05. | 12.06. | 1750 | 37.512 | 2010 |
| 9 | Visp | 2014 | 29.04.-01.05. | 12.05.-15.05. | 04.06.-06.06. | 2820 | 65.028 | 840 |
| 10 | Visp | 2014 | 29.04.-01.05. | 12.05.-15.05. | 04.06.-06.06. | 2050 | 49.834 | 960 |
| 11 | Visp | 2014 | 29.04.-01.05. | 12.05.-15.05. | 04.06.-06.06. | 2540 | 58.902 | 1070 |
| 12 | Visp | 2014 | 29.04.-01.05. | 12.05.-15.05. | 04.06.-06.06. | 2680 | 61.651 | 1280 |
