# Supplementary material for: Effects of forest wildfire on inner-Alpine bird community dynamics
Source: PLoS One. 2019 Apr 24;14(4):e0214644. doi: 10.1371/journal.pone.0214644 (PMC6481801; doi:10.1371/journal.pone.0214644)
Supplement: S2 Table — Several corvid species and all birds of prey were excluded from the analysis. LC = least concern, NT = near-threatened, VU = vulnerable, EN = endangered. (DOCX) [file pone.0214644.s004.docx]

**S2 Table. List of excluded species in all years with Swiss Red list category and Swiss priority status.**

| **English name** | **Latin name** | **Swiss Red list category** | **Swiss priority status** |
| --- | --- | --- | --- |
| Golden Eagle | *Aquila chrysaetos* | VU | no |
| Common Buzzard | *Buteo* *buteo* | LC | no |
| European Honey Buzzard | *Pernis* *apivorus* | NT | no |
| Eurasian Sparrowhawk | *Accipiter* *nisus* | LC | no |
| Northern Goshawk | *Accipiter gentilis* | LC | no |
| Peregrine Falcon | *Falco peregrinus* | NT | no |
| Common Kestrel | *Falco* *tinnunculus* | NT | yes |
| Red-billed Chough | *Pyrrhocorax pyrrhocorax* | EN | no |
| Carrion Crow | *Corvus corone* | LC | no |
| Common Raven | *Corvus corax* | LC | no |

Several corvid species and all birds of prey were excluded from the analysis (LC=least concern, NT=near-threatened, VU=vulnerable, EN=endangered).
