# Supplementary material for: Effects of forest wildfire on inner-Alpine bird community dynamics
Source: PLoS One. 2019 Apr 24;14(4):e0214644. doi: 10.1371/journal.pone.0214644 (PMC6481801; doi:10.1371/journal.pone.0214644)
Supplement: S3 Table — Species highlighted in bold were used for species-specific analyses, while all species were used for community analyses (LC = least concern, NT = near-threatened, VU = vulnerable). (DOCX) [file pone.0214644.s005.docx]

**S3 Table. List of all species found in our study sites in all years with Swiss Red list category and Swiss priority status.**

| **English name** | **Latin name** | **Swiss Red list category** | **Swiss priority status** | **Species recorded in** |
| --- | --- | --- | --- | --- |
| Rock Partridge | *Alectoris graeca* | NT | yes | Leuk only |
| **Black Grouse** | ***Lyrurus tetrix*** | **NT** | **yes** | **Leuk only** |
| Common Woodpigeon | *Columba palumbus* | LC | no | both sites |
| **Common Cuckoo** | ***Cuculus canorus*** | **NT** | **yes** | **both sites** |
| Common Hoopoe | *Upupa epops* | VU | yes | Leuk only |
| **Eurasian Wryneck** | ***Jynx torquilla*** | **NT** | **yes** | **Leuk only** |
| **Eurasian Green Woodpecker** | ***Picus viridis*** | **LC** | **no** | **Leuk only** |
| **Black Woodpecker** | ***Dryocopus martius*** | **LC** | **no** | **both sites** |
| Three-toed Woodpecker | *Picoides tridactylus* | LC | no | Leuk only |
| **Great Spotted Woodpecker** | ***Dendrocopos major*** | **LC** | **no** | **both sites** |
| **Tree Pipit** | ***Anthus trivialis*** | **LC** | **no** | **both sites** |
| Wood Lark | *Lullula arborea* | VU | yes | Leuk only |
| Spotted Flycatcher | *Muscicapa striata* | LC | no | both sites |
| European Pied Flycatcher | *Ficedula hypoleuca* | LC | no | Leuk only |
| **Black Redstart** | ***Phoenicurus ochruros*** | **LC** | **no** | **both sites** |
| **Common Redstart** | ***Phoenicurus phoenicurus*** | **NT** | **yes** | **both sites** |
| **European Robin** | ***Erithacus rubecula*** | **LC** | **no** | **both sites** |
| Rufous-tailed Rock-thrush | *Monticola saxatilis* | LC | no | Leuk only |
| **Common Chiffchaff** | ***Phylloscopus collybita*** | **LC** | **no** | **both sites** |
| **Western Bonelli's Warbler** | ***Phylloscopus bonelli*** | **LC** | **no** | **both sites** |
| Willow Warbler | *Phylloscopus trochilus* | VU | yes | Visp only |
| Wood Warbler | *Phylloscopus sibilatrix* | VU | yes | both sites |
| **Blackcap** | ***Sylvia atricapilla*** | **LC** | **no** | **both sites** |
| Garden Warbler | *Sylvia borin* | NT | no | Leuk only |
| Lesser Whitethroat | *Sylvia curruca* | LC | no | Leuk only |
| Common Whitethroat | *Sylvia communis* | NT | yes | Leuk only |
| **Eurasian Blackbird** | ***Turdus merula*** | **LC** | **no** | **both sites** |
| **Ring Ouzel** | ***Turdus torquatus*** | **VU** | **yes** | **both sites** |
| **Song Thrush** | ***Turdus philomelos*** | **LC** | **no** | **both sites** |
| **Mistle Thrush** | ***Turdus viscivorus*** | **LC** | **no** | **both sites** |
| **Great Tit** | ***Parus major*** | **LC** | **no** | **both sites** |
| **Eurasian Blue Tit** | ***Parus caeruleus*** | **LC** | **no** | **both sites** |
| **Willow Tit** | ***Parus montanus*** | **LC** | **no** | **both sites** |
| **Coal Tit** | ***Parus ater*** | **LC** | **no** | **both sites** |
| **Crested Tit** | ***Parus cristatus*** | **LC** | **no** | **both sites** |
| Firecrest | *Regulus ignicapilla* | LC | no | both sites |
| Goldcrest | *Regulus regulus* | LC | no | both sites |
| **Long-tailed Tit** | ***Aegithalos caudatus*** | **LC** | **no** | **both sites** |
| **Winter Wren** | ***Troglodytes troglodytes*** | **LC** | **no** | **both sites** |
| **Dunnock** | ***Prunella modularis*** | **LC** | **no** | **Leuk only** |
| **Wood Nuthatch** | ***Sitta europaea*** | **LC** | **no** | **both sites** |
| **Eurasian Treecreeper** | ***Certhia familiaris*** | **LC** | **no** | **both sites** |
| Red-backed Shrike | *Lanius collurio* | LC | no | both sites |
| **Eurasian Jay** | ***Garrulus glandarius*** | **LC** | **no** | **both sites** |
| **Spotted Nutcracker** | ***Nucifraga caryocatactes*** | **LC** | **no** | **both sites** |
| **Eurasian Chaffinch** | ***Fringilla coelebs*** | **LC** | **no** | **both sites** |
| European Greenfinch | *Carduelis chloris* | LC | no | Leuk only |
| **European Goldfinch** | ***Carduelis carduelis*** | **LC** | **no** | **both sites** |
| **Alpine Citril Finch** | ***Carduelis citrinella*** | **LC** | **no** | **Leuk only** |
| Eurasian Siskin | *Carduelis spinus* | LC | no | Leuk only |
| **Eurasian Linnet** | ***Carduelis cannabina*** | **NT** | **no** | **Leuk only** |
| Common Redpoll | *Carduelis flammea* | LC | no | Leuk only |
| **European Serin** | ***Serinus serinus*** | **LC** | **no** | **both sites** |
| **Eurasian Bullfinch** | ***Pyrrhula pyrrhula*** | **LC** | **no** | **both sites** |
| **Red Crossbill** | ***Loxia curvirostra*** | **LC** | **no** | **both sites** |
| Yellowhammer | *Emberiza citrinella* | LC | no | Leuk only |
| Cirl Bunting | *Emberiza cirlus* | NT | yes | Leuk only |
| **Rock Bunting** | ***Emberiza cia*** | **LC** | **no** | **both sites** |

Species highlighted in bold were used for species-specific analyses, while all species were used for community analyses (LC=least concern, NT=near-threatened, VU=vulnerable).
