# Supplementary material for: Effects of forest wildfire on inner-Alpine bird community dynamics
Source: PLoS One. 2019 Apr 24;14(4):e0214644. doi: 10.1371/journal.pone.0214644 (PMC6481801; doi:10.1371/journal.pone.0214644)
Supplement: S4 Table — Numbers following a species name distinguish different but equivalent models in terms of AIC. (DOCX) [file pone.0214644.s006.docx]

**S4 Table.** **Species-specific best models calculated with the dredge function, for species with competing best models in terms of AIC.**

| **Species** | **Model factors** | **Estimate** | **Std. error** | **df** | **z-value** | **AICc** |
| --- | --- | --- | --- | --- | --- | --- |
| **Black Grouse 1** | Null model | -5.00 | 2.04 | 2 | -2.45 | **67.9** |
| **Black Grouse 2** | Intercept | -4.67 | 2.05 | 3 | -2.28 | **69.7** |
|  | Forest state | -0.39 | 0.48 | 3 | -0.80 |  |
| **Eurasian Wryneck 1** | Intercept | -6.62 | 1.19 | 5 | -5.57 | **82.5** |
|  | Forest state | -3.31 | 0.40 | 5 | -8.20 |  |
|  | Years after fire | 1.39 | 0.29 | 5 | 4.79 |  |
|  | Years after fire^2^ | -0.07 | 0.02 | 5 | -4.36 |  |
| **Eurasian Wryneck 2** | Intercept | -7.08 | 1.28 | 6 | -5.52 | **83.4** |
|  | Forest state | -1.57 | 1.25 | 6 | -1.25 |  |
|  | Years after fire | 1.47 | 0.30 | 6 | 4.85 |  |
|  | Years after fire^2^ | -0.08 | 0.02 | 6 | -4.43 |  |
|  | Forest state * Years after fire | -0.21 | 0.15 | 6 | -1.38 |  |
| **Eurasian Wryneck 3** | Intercept | -6.85 | 1.23 | 6 | -5.56 | **84.2** |
|  | Forest state | -2.53 | 0.79 | 6 | -3.21 |  |
|  | Years after fire | 1.42 | 0.30 | 6 | 4.82 |  |
|  | Years after fire^2^ | -0.07 | 0.02 | 6 | -4.37 |  |
|  | Forest state * Years after fire^2^ | -0.01 | 0.01 | 6 | -1.04 |  |
| **Tree Pipit 1** | Intercept | -0.60 | 0.70 | 3 | -0.86 | **173.3** |
|  | Forest state | -1.34 | 0.11 | 3 | -11.91 |  |
| **Tree Pipit 2** | Intercept | -1.21 | 0.68 | 5 | -1.78 | **173.6** |
|  | Forest state | -1.34 | 0.11 | 5 | -11.92 |  |
|  | Years after fire | 0.20 | 0.10 | 5 | 2.12 |  |
|  | Years after fire^2^ | -0.01 | 0.01 | 5 | -1.97 |  |
| **Tree Pipit 3** | Intercept | -0.69 | 0.68 | 4 | -1.02 | **174.8** |
|  | Forest state | -1.34 | 0.11 | 4 | -11.91 |  |
|  | Years after fire | 0.02 | 0.02 | 4 | 1.05 |  |
| **Black Redstart 1** | Intercept | 0.00 | 0.33 | 5 | -0.01 | **111.9** |
|  | Forest state | -4.26 | 0.64 | 5 | -6.72 |  |
|  | Years after fire | -0.11 | 0.04 | 5 | -2.90 |  |
|  | Forest state * Years after fire | 0.17 | 0.08 | 5 | 2.13 |  |
| **Black Redstart 2** | Intercept | -0.17 | 0.33 | 4 | -0.52 | **113.6** |
|  | Forest state | -3.18 | 0.29 | 4 | -10.99 |  |
|  | Years after fire | -0.08 | 0.03 | 4 | -2.41 |  |
| **Common Redstart 1** | Intercept | 0.22 | 0.27 | 4 | 0.83 | **149.7** |
|  | Forest state | -3.02 | 0.15 | 4 | -20.07 |  |
|  | Years after fire | 0.04 | 0.02 | 4 | 2.35 |  |
| **Common Redstart 2** | Intercept | -0.07 | 0.35 | 5 | -0.21 | **151.1** |
|  | Forest state | -3.02 | 0.15 | 5 | -20.07 |  |
|  | Years after fire | 0.15 | 0.09 | 5 | 1.58 |  |
|  | Years after fire^2^ | -0.01 | 0.01 | 5 | -1.18 |  |
| **Common Redstart 3** | Intercept | 0.27 | 0.27 | 5 | 0.99 | **151.7** |
|  | Forest state | -3.27 | 0.34 | 5 | -9.62 |  |
|  | Years after fire | 0.03 | 0.02 | 5 | 1.90 |  |
|  | Forest state * Years after fire | 0.03 | 0.04 | 5 | 0.85 |  |
| **Western Bonelli's Warbler 1** | Intercept | 0.34 | 0.54 | 8 | 0.63 | **283** |
|  | Forest state | -0.29 | 0.55 | 8 | -0.52 |  |
|  | Years after fire | -0.35 | 0.17 | 8 | -2.02 |  |
|  | Years after fire^2^ | 0.03 | 0.01 | 8 | 2.94 |  |
|  | Forest state * Years after fire | 0.42 | 0.19 | 8 | 2.23 |  |
|  | Forest state * Years after fire^2^ | -0.03 | 0.01 | 8 | -2.70 |  |
| **Western Bonelli's Warbler 2** | Intercept | -0.54 | 0.40 | 7 | -1.37 | **284.4** |
|  | Forest state | 0.85 | 0.23 | 7 | 3.67 |  |
|  | Years after fire | -0.03 | 0.10 | 7 | -0.27 |  |
|  | Years after fire^2^ | 0.01 | 0.01 | 7 | 1.88 |  |
|  | Forest state * Years after fire^2^ | -0.01 | 0.00 | 7 | -2.34 |  |
| **Western Bonelli's Warbler 3** | Intercept | -0.94 | 0.37 | 6 | -2.54 | **284.6** |
|  | Forest state | 0.99 | 0.33 | 6 | 2.97 |  |
|  | Years after fire | 0.14 | 0.04 | 6 | 3.48 |  |
|  | Forest state * Years after fire | -0.08 | 0.05 | 6 | -1.80 |  |
| **Western Bonelli's Warbler 4** | Intercept | -0.58 | 0.32 | 5 | -1.82 | **284.7** |
|  | Forest state | 0.49 | 0.18 | 5 | 2.70 |  |
|  | Years after fire | 0.09 | 0.03 | 5 | 3.08 |  |
| **Common Chiffchaff 1** | Null model | -1.16 | 0.27 | 2 | -4.29 | **184.1** |
| **Common Chiffchaff 2** | Intercept | -1.79 | 0.47 | 4 | -3.79 | **184.4** |
|  | Years after fire | 0.20 | 0.10 | 4 | 1.97 |  |
|  | Years after fire^2^ | -0.01 | 0.01 | 4 | -1.69 |  |
| **Common Chiffchaff 3** | Intercept | -1.34 | 0.35 | 3 | -3.78 | **184.8** |
|  | Years after fire | 0.03 | 0.03 | 3 | 1.30 |  |
| **Eurasian Blackbird 1** | Intercept | 0.04 | 0.09 | 3 | 0.52 | **220.6** |
|  | Forest state | -0.65 | 0.10 | 3 | -6.36 |  |
| **Eurasian Blackbird 2** | Intercept | -0.09 | 0.15 | 4 | -0.60 | **220.7** |
|  | Forest state | -0.66 | 0.10 | 4 | -6.36 |  |
|  | Years after fire | 0.03 | 0.02 | 4 | 1.66 |  |
| **Ring Ouzel 1** | Intercept | -3.59 | 1.42 | 3 | -2.52 | **124.9** |
|  | Forest state | 0.39 | 0.25 | 3 | 1.55 |  |
| **Ring Ouzel 2** | Null model | -3.26 | 1.39 | 2 | -2.34 | **125.1** |
| **Ring Ouzel 3** | Intercept | -3.75 | 1.37 | 4 | -2.73 | **125.9** |
|  | Forest state | 0.39 | 0.25 | 4 | 1.55 |  |
|  | Years after fire | 0.03 | 0.03 | 4 | 1.27 |  |
| **Ring Ouzel 4** | Intercept | -3.42 | 1.34 | 3 | -2.55 | **125.9** |
|  | Years after fire | 0.03 | 0.03 | 3 | 1.27 |  |
| **Song Thrush 1** | Intercept | -1.99 | 0.21 | 4 | -9.30 | **175.4** |
|  | Forest state | 1.33 | 0.20 | 4 | 6.67 |  |
|  | Years after fire | 0.06 | 0.01 | 4 | 4.45 |  |
| **Song Thrush 2** | Intercept | -1.54 | 0.40 | 5 | -3.83 | **176.5** |
|  | Forest state | 0.85 | 0.41 | 5 | 2.04 |  |
|  | Years after fire | -0.01 | 0.05 | 5 | -0.16 |  |
|  | Forest state * Years after fire | 0.07 | 0.06 | 5 | 1.25 |  |
| **Mistle Thrush 1** | Intercept | -0.55 | 0.09 | 3 | -5.86 | **185.9** |
|  | Years after fire | 0.03 | 0.01 | 3 | 2.32 |  |
| **Mistle Thrush 2** | Null model | -0.43 | 0.11 | 2 | -3.92 | **186.1** |
| **Mistle Thrush 3** | Intercept | -0.42 | 0.13 | 4 | -3.20 | **186.4** |
|  | Forest state | -0.16 | 0.11 | 4 | -1.47 |  |
|  | Years after fire | 0.03 | 0.01 | 4 | 2.24 |  |
| **Mistle Thrush 4** | Intercept | -0.30 | 0.14 | 3 | -2.18 | **186.6** |
|  | Forest state | -0.15 | 0.11 | 3 | -1.39 |  |
| **Mistle Thrush 5** | Intercept | -0.74 | 0.19 | 4 | -3.85 | **187.1** |
|  | Years after fire | 0.10 | 0.06 | 4 | 1.59 |  |
|  | Years after fire^2^ | -0.01 | 0.00 | 4 | -1.15 |  |
| **Mistle Thrush 6** | Intercept | -0.61 | 0.22 | 5 | -2.80 | **187.8** |
|  | Forest state | 0.08 | 0.24 | 5 | 0.32 |  |
|  | Years after fire | 0.05 | 0.03 | 5 | 2.06 |  |
|  | Forest state * Years after fire | -0.03 | 0.03 | 5 | -1.14 |  |
| **Mistle Thrush 7** | Intercept | -0.60 | 0.22 | 5 | -2.81 | **187.9** |
|  | Forest state | -0.16 | 0.11 | 5 | -1.43 |  |
|  | Years after fire | 0.09 | 0.06 | 5 | 1.53 |  |
|  | Years after fire^2^ | 0.00 | 0.00 | 5 | -1.10 |  |
| **Eurasian Blue Tit 1** | Intercept | -4.36 | 0.75 | 4 | -5.80 | **60.1** |
|  | Forest state | -1.48 | 0.52 | 4 | -2.86 |  |
|  | Years after fire | 0.19 | 0.07 | 4 | 2.71 |  |
| **Eurasian Blue Tit 2** | Intercept | -5.61 | 1.32 | 5 | -4.24 | **60.4** |
|  | Forest state | 0.64 | 1.56 | 5 | 0.41 |  |
|  | Years after fire | 0.32 | 0.12 | 5 | 2.66 |  |
|  | Forest state * Years after fire | -0.23 | 0.16 | 5 | -1.49 |  |
| **Crested Tit 1** | Intercept | -1.64 | 0.21 | 3 | -7.88 | **187.1** |
|  | Forest state | 1.90 | 0.20 | 3 | 9.31 |  |
| **Crested Tit 2** | Intercept | -1.55 | 0.21 | 4 | -7.36 | **187.5** |
|  | Forest state | 1.90 | 0.20 | 4 | 9.34 |  |
|  | Years after fire | -0.02 | 0.01 | 4 | -1.75 |  |
| **Long-tailed Tit 1** | Null model | -2.90 | 0.16 | 2 | -17.87 | **113.1** |
| **Long-tailed Tit 2** | Intercept | -3.64 | 0.72 | 4 | -5.08 | **114.8** |
|  | Years after fire | 0.33 | 0.24 | 4 | 1.38 |  |
|  | Years after fire^2^ | -0.03 | 0.02 | 4 | -1.55 |  |
| **Long-tailed Tit 3** | Intercept | -2.95 | 0.38 | 3 | -7.79 | **114.9** |
|  | Years after fire | 0.06 | 0.42 | 3 | 0.14 |  |
| **Winter Wren 1** | Intercept | -0.33 | 0.22 | 7 | -1.45 | **245.9** |
|  | Forest state | -1.12 | 0.15 | 7 | -7.39 |  |
|  | Years after fire | 0.24 | 0.07 | 7 | 3.61 |  |
|  | Years after fire^2^ | -0.03 | 0.01 | 7 | -4.93 |  |
|  | Forest state * Years after fire^2^ | 0.02 | 0.00 | 7 | 5.72 |  |
| **Winter Wren 2** | Intercept | 0.06 | 0.25 | 7 | 0.23 | **247.4** |
|  | Forest state | -1.61 | 0.23 | 7 | -7.07 |  |
|  | Years after fire | 0.08 | 0.07 | 7 | 1.25 |  |
|  | Years after fire^2^ | -0.01 | 0.00 | 7 | -3.15 |  |
|  | Forest state * Years after fire | 0.20 | 0.04 | 7 | 5.64 |  |
| **Dunnock 1** | Intercept | -4.33 | 4.91 | 5 | -0.88 | **138.8** |
|  | Forest state | -0.81 | 0.35 | 5 | -2.36 |  |
|  | Years after fire | -0.17 | 0.05 | 5 | -3.43 |  |
|  | Forest state * Years after fire | 0.17 | 0.05 | 5 | 3.29 |  |
| **Dunnock 2** | Intercept | -4.73 | 4.91 | 6 | -0.96 | **140.3** |
|  | Forest state | -0.34 | 0.22 | 6 | -1.50 |  |
|  | Years after fire | -0.03 | 0.10 | 6 | -0.26 |  |
|  | Years after fire^2^ | -0.01 | 0.01 | 6 | -1.34 |  |
|  | Forest state * Years after fire^2^ | 0.01 | 0.00 | 6 | 3.26 |  |
| **Wood Nuthatch 1** | Intercept | -0.94 | 0.65 | 5 | -1.46 | **140.9** |
|  | Forest state | -0.76 | 0.57 | 5 | -1.32 |  |
|  | Years after fire | -0.29 | 0.11 | 5 | -2.61 |  |
|  | Forest state * Years after fire | 0.30 | 0.11 | 5 | 2.74 |  |
| **Wood Nuthatch 2** | Intercept | -1.91 | 0.56 | 6 | -3.43 | **142** |
|  | Forest state | -0.11 | 0.39 | 6 | -0.27 |  |
|  | Years after fire | 0.13 | 0.13 | 6 | 1.05 |  |
|  | Years after fire^2^ | -0.04 | 0.02 | 6 | -2.35 |  |
|  | Forest state * Years after fire^2^ | 0.03 | 0.01 | 6 | 2.44 |  |
| **Wood Nuthatch 3** | Intercept | -1.18 | 0.68 | 6 | -1.74 | **142.7** |
|  | Forest state | -0.88 | 0.60 | 6 | -1.46 |  |
|  | Years after fire | -0.18 | 0.15 | 6 | -1.14 |  |
|  | Years after fire^2^ | -0.01 | 0.01 | 6 | -1.03 |  |
|  | Forest state * Years after fire | 0.32 | 0.12 | 6 | 2.78 |  |
| **Eurasian**  **Treecreeper 1** | Intercept | -1.17 | 0.48 | 6 | -2.46 | **174.9** |
|  | Forest state | -0.42 | 0.23 | 6 | -1.78 |  |
|  | Years after fire | 0.17 | 0.10 | 6 | 1.75 |  |
|  | Years after fire^2^ | -0.03 | 0.01 | 6 | -3.45 |  |
|  | Forest state * Years after fire^2^ | 0.02 | 0.01 | 6 | 3.52 |  |
| **Eurasian**  **Treecreeper 2** | Intercept | -2.49 | 1.01 | 7 | -2.48 | **175.4** |
|  | Forest state | 1.05 | 0.99 | 7 | 1.06 |  |
|  | Years after fire | 0.66 | 0.34 | 7 | 1.94 |  |
|  | Years after fire^2^ | -0.07 | 0.03 | 7 | -2.47 |  |
|  | Forest state * Years after fire | -0.53 | 0.35 | 7 | -1.53 |  |
|  | Forest state * Years after fire^2^ | 0.06 | 0.03 | 7 | 2.11 |  |
| **Eurasian Chaffinch 1** | Intercept | 0.81 | 0.15 | 5 | 5.32 | **248.9** |
|  | Forest state | 0.47 | 0.15 | 5 | 3.03 |  |
|  | Years after fire | -0.08 | 0.02 | 5 | -3.59 |  |
|  | Forest state * Years after fire | 0.09 | 0.02 | 5 | 4.02 |  |
| **Eurasian Chaffinch 2** | Intercept | 0.69 | 0.16 | 6 | 4.23 | **250.2** |
|  | Forest state | 0.45 | 0.15 | 6 | 2.89 |  |
|  | Years after fire | -0.03 | 0.03 | 6 | -0.94 |  |
|  | Years after fire^2^ | 0.00 | 0.00 | 6 | -1.70 |  |
|  | Forest state * Years after fire | 0.09 | 0.02 | 6 | 4.07 |  |
| **European**  **Goldfinch 1** | Intercept | -0.92 | 0.75 | 6 | -1.24 | **175.9** |
|  | Forest state | -1.81 | 0.60 | 6 | -3.02 |  |
|  | Years after fire | -0.09 | 0.06 | 6 | -1.39 |  |
|  | Forest state * Years after fire | 0.17 | 0.08 | 6 | 2.19 |  |
| **European**  **Goldfinch 2** | Intercept | -1.54 | 0.68 | 4 | -2.27 | **175.9** |
|  | Forest state | -0.70 | 0.29 | 4 | -2.37 |  |
| **European**  **Goldfinch 3** | Intercept | -1.49 | 0.86 | 7 | -1.72 | **177.7** |
|  | Forest state | -1.85 | 0.61 | 7 | -3.05 |  |
|  | Years after fire | 0.12 | 0.20 | 7 | 0.61 |  |
|  | Years after fire^2^ | -0.01 | 0.01 | 7 | -1.10 |  |
|  | Forest state * Years after fire | 0.17 | 0.08 | 7 | 2.23 |  |
| **Alpine Citril Finch 1** | Intercept | -3.12 | 4.74 | 5 | -0.66 | **115.2** |
|  | Forest state | -1.11 | 0.17 | 5 | -6.60 |  |
|  | Years after fire | -0.49 | 0.15 | 5 | -3.35 |  |
|  | Years after fire^2^ | 0.03 | 0.01 | 5 | 2.63 |  |
| **Alpine Citril Finch 2** | Intercept | -2.82 | 4.68 | 6 | -0.60 | **115.4** |
|  | Forest state | -1.62 | 0.35 | 6 | -4.61 |  |
|  | Years after fire | -0.53 | 0.15 | 6 | -3.57 |  |
|  | Years after fire^2^ | 0.02 | 0.01 | 6 | 2.53 |  |
|  | Forest state * Years after fire | 0.08 | 0.05 | 6 | 1.64 |  |
| **Alpine Citril Finch 3** | Intercept | -2.97 | 4.69 | 6 | -0.63 | **115.4** |
|  | Forest state | -1.37 | 0.23 | 6 | -5.94 |  |
|  | Years after fire | -0.48 | 0.15 | 6 | -3.25 |  |
|  | Years after fire^2^ | 0.02 | 0.01 | 6 | 2.11 |  |
|  | Forest state * Years after fire^2^ | 0.01 | 0.00 | 6 | 1.62 |  |
| **Eurasian Linnet 1** | Intercept | -4.14 | 3.00 | 3 | -1.38 | **96.8** |
|  | Forest state | -1.13 | 0.25 | 3 | -4.48 |  |
| **Eurasian Linnet 2** | Intercept | -4.02 | 3.17 | 4 | -1.27 | **98.2** |
|  | Forest state | -1.13 | 0.25 | 4 | -4.48 |  |
|  | Years after fire | -0.04 | 0.04 | 4 | -1.08 |  |
| **European Serin 1** | Intercept | 0.33 | 0.38 | 6 | 0.86 | **141.9** |
|  | Forest state | -5.09 | 0.61 | 6 | -8.31 |  |
|  | Years after fire | -0.01 | 0.14 | 6 | -0.06 |  |
|  | Years after fire^2^ | -0.03 | 0.01 | 6 | -2.21 |  |
|  | Forest state * Years after fire | 0.53 | 0.11 | 6 | 5.00 |  |
| **European Serin 2** | Intercept | -0.14 | 0.41 | 6 | -0.35 | **143.8** |
|  | Forest state | -3.63 | 0.35 | 6 | -10.26 |  |
|  | Years after fire | 0.20 | 0.17 | 6 | 1.22 |  |
|  | Years after fire^2^ | -0.05 | 0.02 | 6 | -2.82 |  |
|  | Forest state * Years after fire^2^ | 0.04 | 0.01 | 6 | 4.46 |  |
| **Eurasian Bullfinch 1** | Intercept | -0.81 | 0.17 | 3 | -4.75 | **194.7** |
|  | Forest state | -0.49 | 0.15 | 3 | -3.28 |  |
| **Eurasian Bullfinch 2** | Intercept | -0.71 | 0.22 | 4 | -3.22 | **196.3** |
|  | Forest state | -0.49 | 0.15 | 4 | -3.26 |  |
|  | Years after fire | -0.02 | 0.02 | 4 | -0.99 |  |
| **Red Crossbill 1** | Intercept | -4.54 | 1.35 | 4 | -3.35 | **182.3** |
|  | Forest state | 2.54 | 0.55 | 4 | 4.59 |  |
| **Red Crossbill 2** | Intercept | -6.18 | 1.42 | 6 | -4.37 | **182.8** |
|  | Forest state | 2.47 | 0.47 | 6 | 5.21 |  |
|  | Years after fire | 0.67 | 0.28 | 6 | 2.41 |  |
|  | Years after fire^2^ | -0.04 | 0.02 | 6 | -2.52 |  |
| **Rock Bunting 1** | Intercept | 0.65 | 0.50 | 4 | 1.29 | **203.6** |
|  | Forest state | -3.02 | 0.20 | 4 | -14.99 |  |
| **Rock Bunting 2** | Intercept | 0.19 | 0.64 | 6 | 0.29 | **205** |
|  | Forest state | -3.04 | 0.18 | 6 | -16.95 |  |
|  | Years after fire | 0.21 | 0.14 | 6 | 1.51 |  |
|  | Years after fire^2^ | -0.02 | 0.01 | 6 | -1.81 |  |
| **Rock Bunting 3** | Intercept | 0.84 | 0.58 | 5 | 1.45 | **205.1** |
|  | Forest state | -3.03 | 0.19 | 5 | -15.74 |  |
|  | Years after fire | -0.04 | 0.03 | 5 | -1.14 |  |

Numbers following a species name distinguish different but equivalent models in terms of AIC.
