# Supplementary material for: Effects of forest wildfire on inner-Alpine bird community dynamics
Source: PLoS One. 2019 Apr 24;14(4):e0214644. doi: 10.1371/journal.pone.0214644 (PMC6481801; doi:10.1371/journal.pone.0214644)
Supplement: S5 Table — Model selection table for species richness. (DOCX) [file pone.0214644.s007.docx]

**S5 Table. Model selection tables for analyses, where competing best models were found.**

| Intercept | Foreststate | df | logLik | AICc | delta | weight |
| --- | --- | --- | --- | --- | --- | --- |
| 3.49 |  | 2 | -103.69 | 211.7 | 0 | 0.43 |
| 3.44 | + | 3 | -102.94 | 212.6 | 0.88 | 0.28 |

Model selection table for species richness
