# Supplementary material for: Effects of forest wildfire on inner-Alpine bird community dynamics
Source: PLoS One. 2019 Apr 24;14(4):e0214644. doi: 10.1371/journal.pone.0214644 (PMC6481801; doi:10.1371/journal.pone.0214644)
Supplement: S6 Table — a Model selection table for species abundance. b Model selection table for priority analysis with index. (DOCX) [file pone.0214644.s008.docx]

**S6 Table. Model selection tables for analyses, where no competing best models were found.**

a

| Intercept | Forest-state | years after fire | years after fire^2^ | Forest state* years after fire | df | logLik | AICc | delta | weight |
| --- | --- | --- | --- | --- | --- | --- | --- | --- | --- |
| 21.68 | + |  |  |  | 4 | -100.29 | 209.9 | 0 | 0.61 |
| 20.43 | + | 0.24 |  |  | 5 | -100.31 | 212.6 | 2.75 | 0.15 |
| 15 | + | 2.35 | -0.14 |  | 6 | -99.21 | 213.3 | 3.44 | 0.11 |
| 22.27 | + | -0.09 |  | + | 6 | -99.55 | 214 | 4.13 | 0.08 |
| 16.84 | + | 2.02 | -0.14 | + | 7 | -98.39 | 214.8 | 4.92 | 0.05 |

Model selection table for species abundance

b

| Intercept | Priority status | years after fire | years after fire^2^ | Priority status* years after fire | df | logLik | AICc | delta |
| --- | --- | --- | --- | --- | --- | --- | --- | --- |
| -1.07 | + | -0.07 |  | + | 7 | -1075.53 | 2165.3 | 0 |
| -1.42 | + |  |  |  | 5 | -1079.43 | 2169 | 3.69 |
| -1.03 |  |  |  |  | 4 | -1082.62 | 2173.3 | 8.04 |
| -1.38 | + | 0.05 | -0.01 | + | 8 | -1079.1 | 2174.5 | 9.2 |
| -1.3 | + | -0.03 |  |  | 6 | -1081.78 | 2175.7 | 10.43 |

Model selection table for priority analysis with index
