# Supplementary material for: Effects of forest wildfire on inner-Alpine bird community dynamics
Source: PLoS One. 2019 Apr 24;14(4):e0214644. doi: 10.1371/journal.pone.0214644 (PMC6481801; doi:10.1371/journal.pone.0214644)
Supplement: S7 Table — a Best model for Red list analysis with index after model averaging. b Best model for Red list analysis with territories after model averaging. c Best model for priority analysis with territories after model averaging. (DOCX) [file pone.0214644.s009.docx]

**S7 Table. Averaged best models for Red list and priority analyses, calculated only for analyses with competing best models from Tables 2-4.**

a

|  | Estimate | Std. Error | Adjusted SE | z-value |
| --- | --- | --- | --- | --- |
| Intercept | -1.31 | 0.59 | 0.59 | 2.23 |
| Red list category | 1.69 | 1.66 | 1.67 | 1.02 |
| Years after fire | -0.07 | 0.03 | 0.03 | 2.58 |
| Red list category* years after fire | 0.27 | 0.07 | 0.07 | 3.67 |

Best model for Red list analysis with index after model averaging.

b

|  | Estimate | Std. Error | Adjusted SE | z-value |
| --- | --- | --- | --- | --- |
| Intercept | -2.63 | 0.43 | 0.43 | 6.12 |
| Forest state | -0.36 | 0.14 | 0.14 | 2.59 |
| Red list category | -1.43 | 0.95 | 0.95 | 1.51 |
| Years after fire | 0.05 | 0.05 | 0.05 | 1.1 |
| Years after fire^2^ | -0.01 | 0.003 | 0.003 | 1.98 |
| Red list category *forest state | -0.91 | 0.34 | 0.34 | 2.68 |
| Red list category* years after fire | 0.17 | 0.08 | 0.08 | 2.13 |
| Red list category* years after fire^2^ | 0.002 | 0.01 | 0.01 | 0.19 |
| Forest state* years after fire | 0.04 | 0.04 | 0.04 | 1.16 |
| Forest state* years after fire^2^ | 0.003 | 0.003 | 0.003 | 1.01 |
| Red list category* forest state * years after fire | -0.11 | 0.05 | 0.05 | 2.38 |
| Red list category* forest state * years after fire^2^ | -0.01 | 0.003 | 0.003 | 2.35 |

Best model for Red list analysis with territories after model averaging.

c

|  | Estimate | Std. Error | Adjusted SE | z-value |
| --- | --- | --- | --- | --- |
| Intercept | -2.86 | 0.43 | 0.43 | 6.68 |
| Forest state | -0.35 | 0.13 | 0.13 | 2.6 |
| Priority status | -0.32 | 1.09 | 1.09 | 0.29 |
| Years after fire | 0.06 | 0.05 | 0.05 | 1.38 |
| Years after fire^2^ | -0.01 | 0.003 | 0.003 | 2.19 |
| Priority status * forest state | -0.91 | 0.36 | 0.36 | 2.55 |
| Priority status * years after fire | 0.15 | 0.07 | 0.07 | 2.06 |
| Priority status * years after fire^2^ | 0.01 | 0.01 | 0.01 | 1.49 |
| Forest state * years after fire | 0.04 | 0.05 | 0.05 | 0.79 |
| Forest state * years after fire^2^ | 0.003 | 0.003 | 0.003 | 1.25 |
| Priority status* forest state * years after fire | -0.12 | 0.05 | 0.05 | 2.50 |
| Priority status* forest state * years after fire^2^ | -0.01 | 0.003 | 0.003 | 2.52 |

Best model for priority analysis with territories after model averaging.
